# An Augmented Reality Game for Helping Elderly to Perform Physical Exercises at Home

**DOI:** 10.1007/978-3-030-58796-3_28

**Published:** 2020-08-10

**Authors:** Anna Nishchyk, Wim Geentjens, Alejandro Medina, Marie Klein, Weiqin Chen

**Affiliations:** 8 Institute Integriert Studieren, JKU Linz, Linz, Austria; 9 Jack Baskin School of Engineering, UC Santa Cruz, Santa Cruz, CA USA; 10 Dipartimento di Meccanica, Politecnico di Milano, Milan, Italy; 11 Support Centre for Students with Special Needs, Masaryk University Brno, Brno, Czech Republic; Oslo Metropolitan University, St. Olavs Plass, 0130 Oslo, Norway

**Keywords:** Augmented reality, Exergame, Fall prevention, Elderly, Physical exercises

## Abstract

People are living longer nowadays. Unfortunately, this positive tendency is marred by various age-related health issues, which people experience. Falling is one of the most serious and common of them. Falls negatively influences elderly’ everyday living and significantly decreases quality of their life. Physical exercises is a proven method for preventing falls. However, it is only effective when training is regular and exercise techniques are correct. This paper presents a prototype of an augmented reality exergame for elderly people to perform physical exercise at home. The research is focusing on developing a solution for both above-mentioned issues: augmentation with Microsoft Kinect and various sensors assists in creating a safe game environment, which can helps to perform exercises with right technique; gamification elements contribute to users’ motivation to train regularly. A user-centered design approach was adopted to guide the design and development iterative process. User testing of the first prototype was performed and demonstrated positive attitudes from participants. Feedback from user testing will be used for the next development iterations.

## Background

Population around the world is rapidly ageing and the numbers are only increasing each year [12]. Unfortunately, with age the majority of elderly people are losing muscle mass and strength, which could lead to limited mobility, decrease in postural control and increase of risk of falling and related injuries [5, 6]. Falling is one of the most common and serious age-related issues, which negatively affects the health of elderly and their well-being. About 1/3 of community dwelling older people over 65 experience a fall annually [4, 6].

Many research have been focusing on different fall prevention methods [4, 6, 9, 10]. A number of articles demonstrated that regular physical exercises can help to reduce the amount of falls and improve health conditions [3, 4, 10]. Training is recognized as an effective and cost-efficient fall prevention method [3–5]. However, exercises also have risks, related to wrong technique and wrong speed of the training [7]. It could also be challenging to perform exercises regularly, especially for elderly people, who have experienced a fall before and developed a fear of falling. According to the literature, up to 24-55% of seniors have a fear of falling [8].

The use of different interventions, such as video exercising games, virtual and augmented reality games as a method of physical exercise “delivering” has shown its effectiveness based on a number of research studies [10, 11]. Literature have shown that variety of “exergames” can increase motivation to exercise as well as level of enjoyment for different users, including elderly people [10, 11]. Modern technologies such as sensors and other various input devices have made it possible to determinate a user’s exercise performance and show it to the user through a usable interaction interface [11].

However, most of the tested interventions have limitations for the aged generation related to different factors, including a lack of accessibility and usability for people with low technology tolerance, low safety level, etc. [11]. Most of the mentioned interventions are commercial and were not designed to the specific purpose of helping elderly people to perform physical exercises [11].

There is a lack of exergames intervention, which are specifically designed and developed for elderly users. The goal of this research is to create a system with two major focuses: first, to create a safe game environment, which can help to perform exercises with right techniques and reduce risk of traumatic mistakes; second, the elements of the gamification and the game’s narrative can motivate users to do the training regularly. Additionally, the interface of the game should be accessible and usable for elderly people.

One of the interventions, which has been investigated before and demonstrated its effectiveness for fall prevention, is augmented reality [1]. Augmented Reality is a technology, which presents a combination of artificial and real elements (aligned with each other) in a real environment; it is three-dimensional, interactive and working in a real time [13]. Augmented reality is highly useful for training purposes, especially due to its ability to create contextual situational experience [15].

The end goal of the research is to demonstrate how augmented reality game, specifically developed for our target group, could help elderly to perform physical exercises, improve their health conditions and, as a result, prevent from falls, which could significantly enhance their quality of life. In terms of this research, our target users will be elderly people (any person over 60), anyone with limited mobility or a person who is prone to lose muscle mass easier.

## Methods

Since the focus of the research is to create a system specifically designed and developed for a certain user group, User-centered design approach has been chosen. This approach helps to focus on the needs of potential users, gain deeper understanding of the issues which they have and clarify how to create the product which they could get maximum benefit of [2].

The design and development process will consist of several iterations with user testing at the end of each iteration. For now, the first iteration is finished, the results of it will be presented in this paper.

## Design and Development

In order to start the design of the first prototype, various recommendations on how to design for elderly users were investigated. To represent the research’s target group and to better understand the need of our target users, two personas were created in the beginning of the design process.

Based on previous research, similar systems and created personas, the following requirements have been defined: creating an indoor solution, which could be used at home to provide safeness and confidence (performing exercises in the familiar environment increases the level of safety); a user should be able to play the game alone, without additional supervision or/and a partner (elderly people often live alone).

Three important components were considered for the design and development. First, physical exercises, which will be chosen as the exercises “packed” in the game shell, should be suitable for the research and its target group. Second, hardware, including sensors, main purpose of which is to make sure the participants’ safety (that the exercises are performed with the right technique). Third, the narrative of the game, its levels and achievements, which will motivate a user to play the game and perform the exercises regularly.

**Fig. 1.**
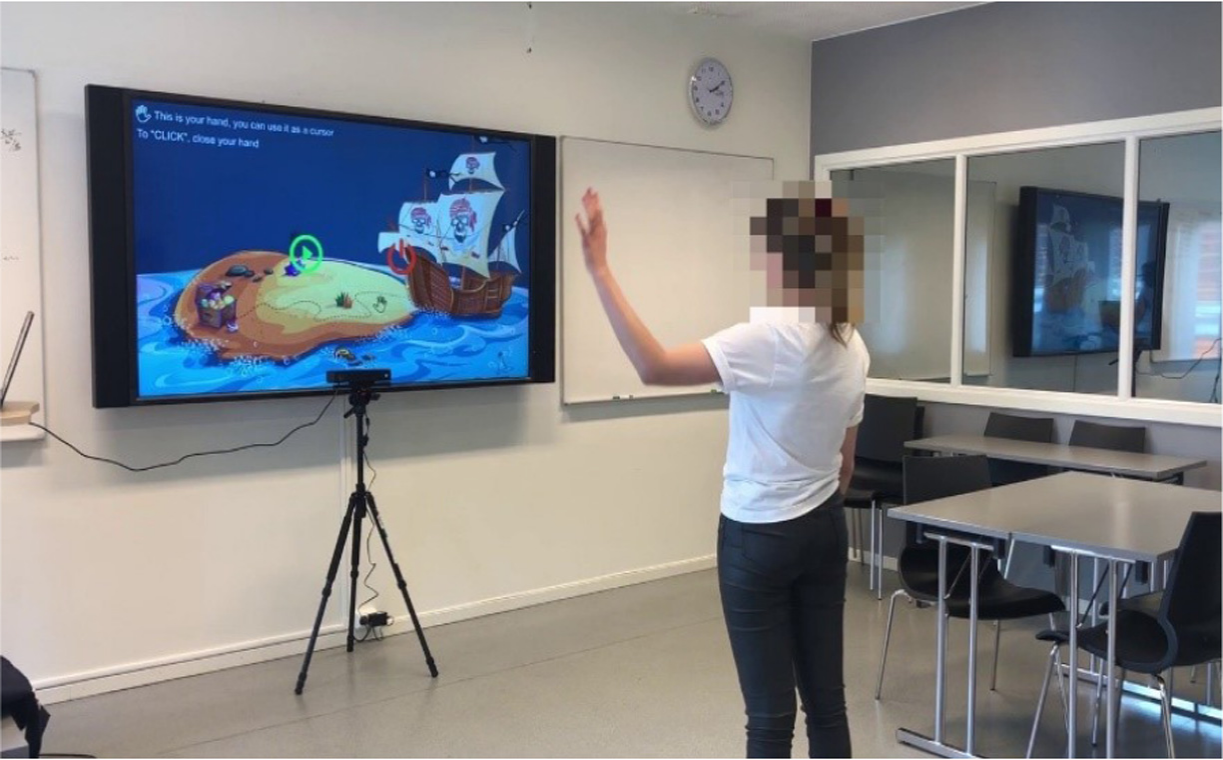
Set-up of the game.

The solution consists of a user-friendly adventure game, created for elderly people using Augmented Reality concept. The augmentation was created by the use of Kinect camera, additional sensors and Arduino. The game has a plug and play solution and well-explained interface (see Fig. 1).

### Exercises

The chosen exercises program is a low-intensity resistance training program with slow movement (LST method) developed by Prof. Ishii [14]. It helps to increase muscle mass and strength, and it is effective and safe for elderly people, which can help to avoid some possible injuries [14]. These exercises are recommended by The National Health Service (the publicly funded healthcare system of the United Kingdom), as suitable for elderly people.

The game is about a pirate and his parrot walking on an island trying to look for a treasure chest. During their trip, they have to pass different challenges before finding the treasure chest. To pass different level of difficulty, a user needs to perform different physical exercises, which fits in the narrative of the game (for examples, “hand swings” exercises allows the parrot to fly, etc.).

### Game Narrative

The game idea aims to engage a user and create a possibility to add new scenarios with new types of exercises easily. It also allows using different methods of motivation like score, rewards, unlockable levels, etc.

**Fig. 2.**
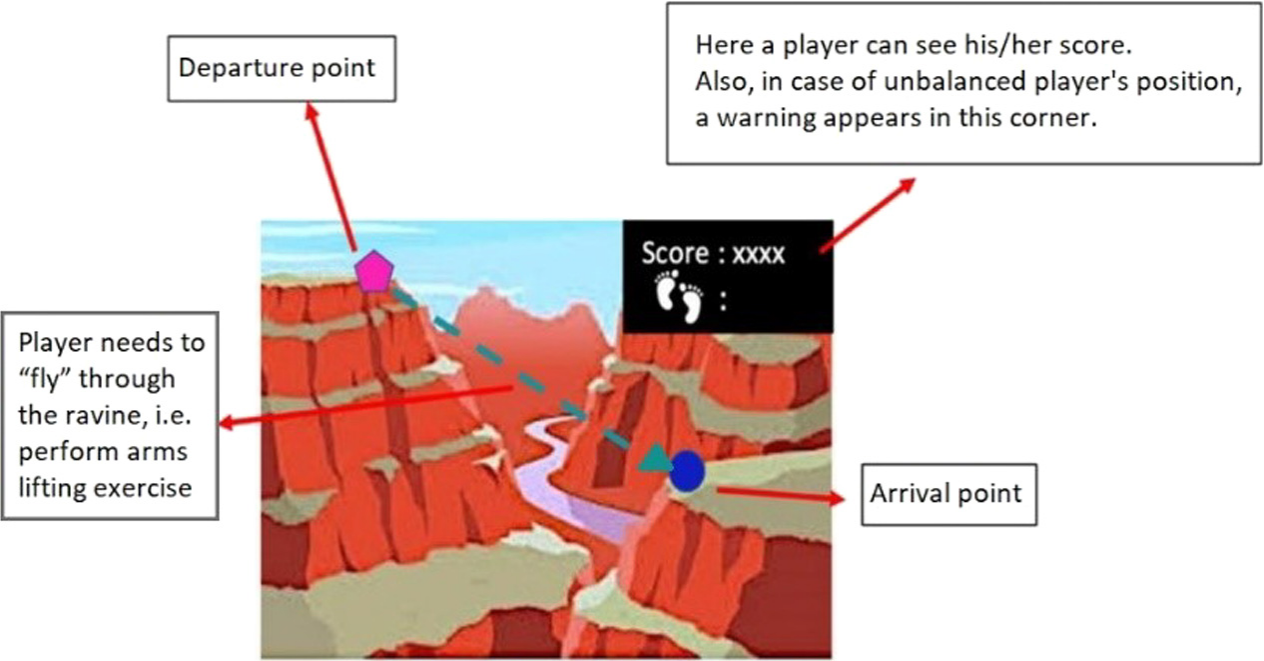
Mock-up of second task.

The game consists of set of missions (tasks). Completing each task allows the player to move forward from point A to point B in the game narrative scenario. To complete each task the player needs to perform a particular physical exercise. The game scenario was designed in a way which helps logically fit each physical exercise into game narrative. During the first game development iteration, presented in this paper, two tasks were developed. Figure 2 shows a mock-up of one of the tasks.

**Fig. 3.**
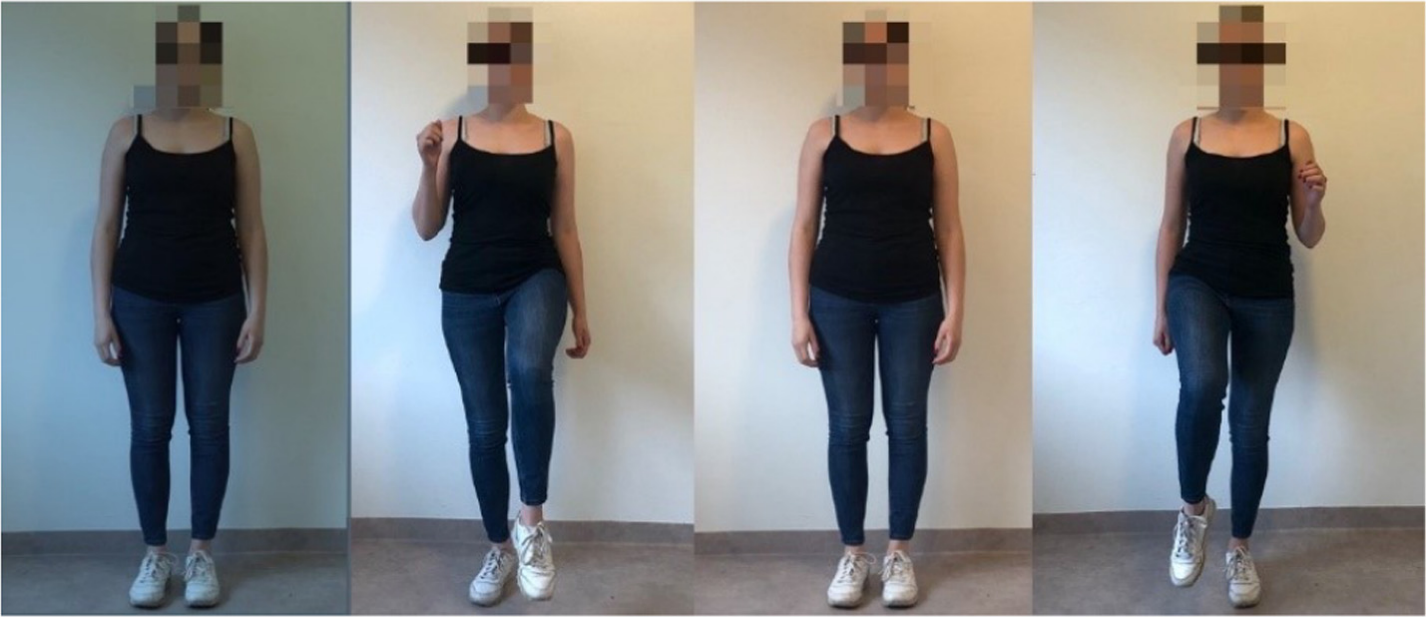
A demonstration of the first exercise.

The first task is to climb a staircase in order to start the journey and leave hometown. The exercise the player needs to perform is to walk with high knees on a spot to imitate staircase climbing (see Fig. 3).

**Fig. 4.**
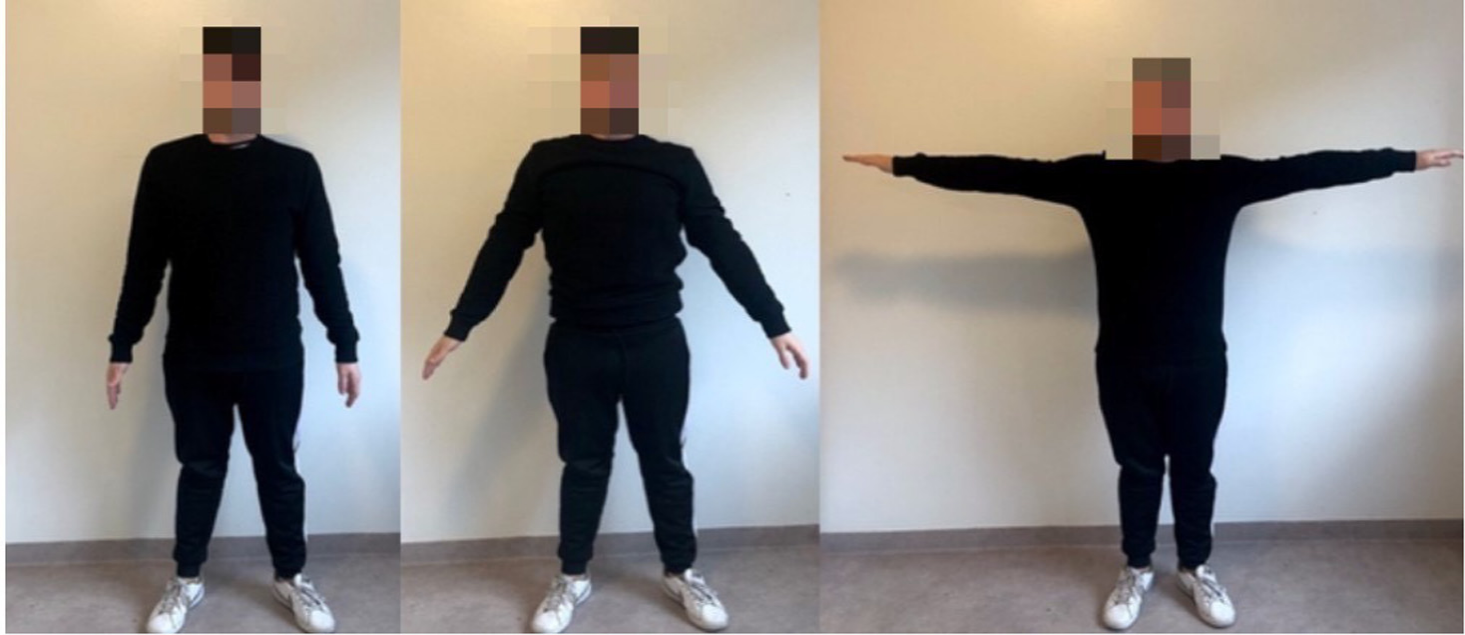
A demonstration of second exercise.

The next game stop is located on top of a mountain in front of a ravine, so the player needs to “fly” through it as a parrot. To “fly” the player needs to repetitively lift arms to shoulder height (parallel to the ground) and keep elbows straight and then lower arms slowly to starting position (see Fig. 4).

Two more exercises were selected and scripted into game tasks’ scenario, but have not been developed yet.

### Hardware

#### Kinect.

Different technologies have been investigated in order to find the best solution for the research. The final choice was Microsoft Kinect. It is a device with a digital camera, which helps with the posture recognition. The technology was used to recognize player’s body, map it as a simple skeleton and distinguish the positions of the body during exercising. Kinect is an open source technology and Microsoft offers a well-documented Software Development Kit for it. A large developers’ community has been using the technology. Much useful information is available.

#### Arduino.

**Fig. 5.**
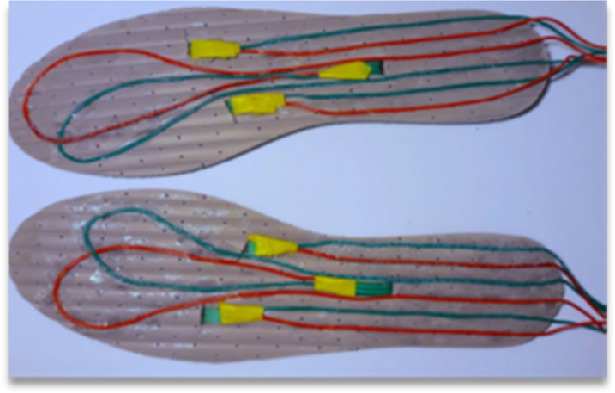
Front of the foot sensor insole with the connected sensors.

**Fig. 6.**
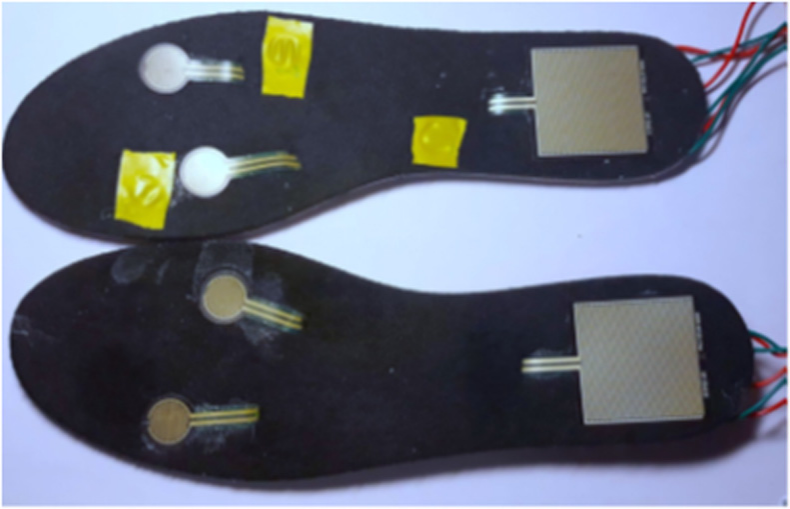
Back of the foot sensor insole with the connected sensors.

Making a safe environment was established as the main priority for the research. A part of the functions is to monitor balance, heart rate and fall detection. For this purpose, a wristband and pressure foot sensors has been designed. Based on the foot sensor design its prototype was developed (see Fig. 5 and Fig. 6).

Open-source electronic microcontroller board and prototyping platform Arduino was used to prototype foot sensors. Several round and square pressure sensors were placed on different parts of insoles. Through simple serial Bluetooth modules the system gets data from foot pressure sensors. Then in real time the program calculates each foot “sum” and “average” pressure and compares right and left foot numbers. If the difference in average is more than 15%, a real time warning appears on the screen and notifies the player that his/her position is unbalanced.

## User Testing and Results

The prototype was tested with three participants. The age of participants ranged from 43 to 62 years old. Two of them were men and one was a woman. One of the participants had shoulder mobility problem, another had issues with his lower back. Although the main research target group is elderly, it was decided to include 43-years old participant into user testing due to his mobility issue and lack of physical exercises on a daily basis.

In order to collect demographic information about the participant, an interview was conducted before user testing procedure. The participants were then asked to turn on the system and play the game following the instructions on the screen. The users’ interaction with the game was observed by researchers in order to identify issues experienced during the process. After the user testing, post-testing interview was conducted to collect participants’ feedback and understand their attitude towards the game.

All the participants managed to finish playing the whole game. The overall feedback was positive. All of them stated that the game was comfortable to play, and the speed and the length of the explanatory videos were correct to understand the exercises. The participants also reported that the additional information collected through the sensors was very useful.

The participants have also provided some feedback, which will be used for the next iteration of the development. For instance, all the participants stated that the narrative of the game is interesting and engaging, however more themes could be added in order to add a variety to the exercising experience. One of the participant claimed that the length and speed of the videos are suitable, but the possibility to skip the video should be available, especially considering that the game could be repeated by a user many times. The participants commented that the game could be more motivating, so it would be important to add more motivation methods (score, rewards, unlockable levels, etc.). One of the participants also emphasized that a few times during the game when he made a mistake, he did not receive feedback. So more feedback from the game to a user should be added in order to increase the usability of the system.

## Conclusion and Future Work

The research aimed to create a safe exercising environment with a game narrative to motivate elderly users to exercise regularly and correctly. The first prototype has been developed with a few basic exercises as a proof of concept, but has a possibility of extension during the next development iterations.

The results of the first prototype testing have shown that the game has potential to achieve the goal of the research. The participants have shown a positive attitude towards the prototype. They have also provided some useful feedback, which would help with further development. Although the game is designed for home use, it may also be used in health institutions, for instance, in nursing homes.

In the next development iterations, the prototype will be developed further taking into consideration the user testing results. It is planned to involve more users in next user testing iterations. After the last iteration, a longitudinal study will be conducted to investigate the effect of the exergame on elderly health conditions and its effectiveness as a fall prevention intervention.
